# Chiral Amplification through the Interplay of Racemizing Conditions and Asymmetric Crystal Growth

**DOI:** 10.1021/jacs.2c10584

**Published:** 2022-12-19

**Authors:** Sjoerd W. van Dongen, Imane Ahlal, Michel Leeman, Bernard Kaptein, Richard M. Kellogg, Iaroslav Baglai, Willem L. Noorduin

**Affiliations:** † AMOLF, Science Park 104, 1098 XGAmsterdam, The Netherlands; ‡ Symeres, Kadijk 3, 9747 ATGroningen, The Netherlands; § InnoSyn BV, Urmonderbaan 22, 6167 RDGeleen, The Netherlands; ∥ Van ‘t Hoff Institute for Molecular Sciences, University of Amsterdam, Science Park 904, 1098 XHAmsterdam, The Netherlands

## Abstract

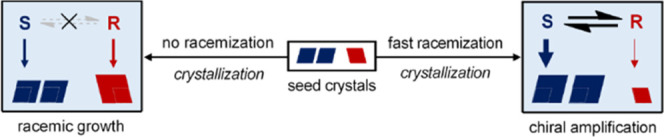

Amplification of enantiomeric excesses (ee) is routinely observed during chiral crystallization of conglomerate crystals for which the enantiomers undergo racemization in solution. Although routes comprising a combination of crystal growth and dissolution are frequently used to obtain enantiopure molecules, crystal growth by itself has rather been considered as a source of enantiomeric erosion and discounted as a potential source of enantiomeric amplification. Counterintuitively, we here demonstrate striking enantiomeric amplification during crystal growth for clopidogrel and *tert*-leucine precursors. Based on a mechanistic framework, we identify that the interplay between racemization and crystal growth rates elicits this surprising effect. The asymmetric amplification of the solid-phase ee can be enhanced by increasing the mass of grown material relative to the product such that small amounts of seeds of only 60% ee already result in virtually exclusive growth of the majority phase. These results impact our understanding of asymmetric amplification mechanisms during crystallization and offer a tangible basis for practical production of enantiopure molecules.

## Introduction

Asymmetric amplification phenomena are of fundamental interest for understanding the emergence of enantiopure building blocks for the origin of life. Moreover, they offer practical routes for synthesis of essential molecules such as agricultural compounds and pharmaceuticals.^1−18^ Amplification has been observed in catalysis, in which the chiral product exhibits a larger enantiomeric excess (ee) than the enantiopurity of the catalysts.^3,19^ In the unique case of the Soai reaction, the reaction product even feeds back to catalyze its own formation, thus resulting in autocatalytic amplification.^3^ In contrast, amplification of ee is routinely observed during crystallization processes when enantiomers undergo racemization in solution while crystallizing in separate crystals (so-called conglomerates).^16,20−33^ In particular, slurries of left- and right-handed enantiomorphic crystals can convert into an enantiopure phase via continuous growth and dissolution using, for instance, temperature gradient deracemization,^34^ temperature cycling-induced deracemization (TCID),^35−42^ or attrition-enhanced deracemization (Viedma ripening).^25,27,29,43−46^ Already, these deracemization processes have been demonstrated for a wide range of molecules, including precursors of agricultural compounds and blockbuster pharmaceuticals.^27,42,44,46−51^ Even though there is still debate over the details of the underlying mechanism in these remarkable processes,^52^ there is a general consensus that the interplay between growth and dissolution is essential for achieving enantioenrichment through crystallization.^30,37,40,53−56^

Indeed, it is not obvious if, and how, merely growth of crystals can lead to any form of enantioenrichment. To understand why this is non-trivial, we consider a population of left and right-handed conglomerate seed crystals that are in contact with a supersaturated racemizing solution. Based on classical crystal growth theory, there is a tacit understanding that each crystal—either left- or right-handed—has a prima facie equal probability to incorporate molecular building blocks from the racemizing solution. Consequently, it is believed that during crystal growth, the masses of both crystal populations would grow proportional to their population size. The initial enantiomeric excess of the solid phase thus remains preserved, and no enantioenrichment is expected. Moreover, racemization is not infinitely fast and perturbations of the supersaturated solution can lead to nucleation. Both of these phenomena favor the minority enantiomer and therefore are expected to result in erosion instead of preservation—let alone amplification—of the chiral purity. Hence, motivated by arguments along these lines of thought, crystal growth is generally considered as a source of erosion of ee and has therefore been discounted as a potential route for chiral amplification.

**Figure 1:**
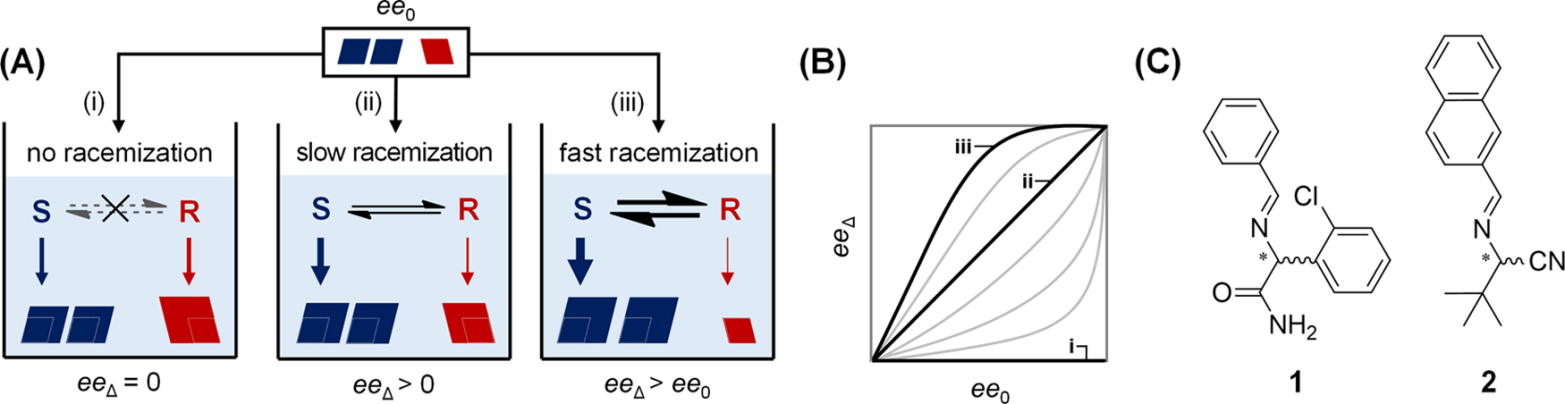
Mechanistic framework for amplification of the solid-phase ee during crystal growth from a supersaturated racemic solution. (A) Balance between the crystallization and racemization rates determines if (i) erosion, (ii) consolidation, or (iii) amplification of the initial enantiomeric excess ee_0_ of the seed crystals occurs: the solute resource pool is limited by the relative racemization rate. (B) ee of the material deposited onto the seed crystals during growth (ee_Δ_) as a function of the ee of the seed crystal ee_0_ for the different scenarios. (C) Racemizable conglomerates **1** and **2** used for demonstrating the mechanistic framework.

Contrary to this line of reasoning, we here experimentally demonstrate that large and systematic chiral amplification can occur during crystal growth such that even seed crystals with low enantiopurity can yield final products with high enantiomeric purity. Using precursors of clopidogrel and *tert*-leucine (Figure 1C) as model compounds, which are known to form conglomerates,^47,49^ we demonstrate that the interplay between the crystal growth rate and racemization rate can create an asymmetric form of crystal growth such that the majority phase grows faster than the minority phase (Figure 1). We show that this surprising imbalance in the growth rates can give rise to amplification of the solid-phase ee during crystallization. These results impact our fundamental understanding of crystal growth, the practical production of chiral molecules, and the discussion on the origin of homochirality.

## Results and Discussion

To guide the experiments, we define a framework to understand how the interplay between the crystallization rate and racemization rate can lead to this counterintuitive asymmetric amplification (Figure 1). We consider seeding a clear supersaturated solution with crystals of low enantiomeric purity (ee_0_) for three scenarios: (i) no racemization, (ii) relatively slow racemization compared to crystallization, and (iii) relatively fast racemization (Figure 1A). We define the enantiomeric excess of the material deposited during growth onto the seed crystals as ee_Δ_. When no racemization is present, we expect erosion—essentially dilution—of the initial enantiomeric excess (ee_Δ_ = 0), since equal amounts of both enantiomers are deposited, maintaining the eutectic composition in solution (ee_eu_ = 0) typical for conglomerate systems. When racemization is initiated, the enantiomers in solution become an increasingly common resource pool available to both populations of enantiomorphic crystals. For slow racemization, an initial enantiomeric excess can potentially be consolidated during growth (ee_Δ_ = ee_0_): the balancing of racemization and crystallization rates allows for the timely replenishment of the faster-growing major enantiomer so that both crystal populations can grow proportionally to their initial composition. Indeed, the extent of amplification is racemization rate limited. When racemization is much faster than crystallization, the supply of major enantiomer is not limiting, and the balance can tip over: the faster growing major enantiomer draws away even more minor enantiomer to lower the supersaturation as fast as possible such that the initial enantiomeric excess is amplified (ee_Δ_ > ee_0_). Visualizing ee_Δ_ for different starting ee_0_’s (Figure 1B),^57^ this framework thus suggests that balancing racemization and crystallization rates can result in either erosion, consolidation, or amplification of ee_0_.

To demonstrate this experimentally, we study the growth of enantiomerically enriched seed crystals in contact with a supersaturated racemic solution in the absence and presence of a racemization catalyst using the compound 2-(benzylideneamino)-2-(2-chlorophenyl)acetamide (**1**, Figure 1C). This precursor to the cardiovascular drug Plavix (clopidogrel) is a conglomerate and can be easily racemized with tunable rate using the organic base 1,8-diazabicyclo[5.4.0]undec-7-ene (DBU) in acetonitrile. This compound has been used extensively to study both Viedma ripening and TCID, making **1** ideally suitable to study growth under racemizing conditions.^40,47,58,59^

**Figure 2:**
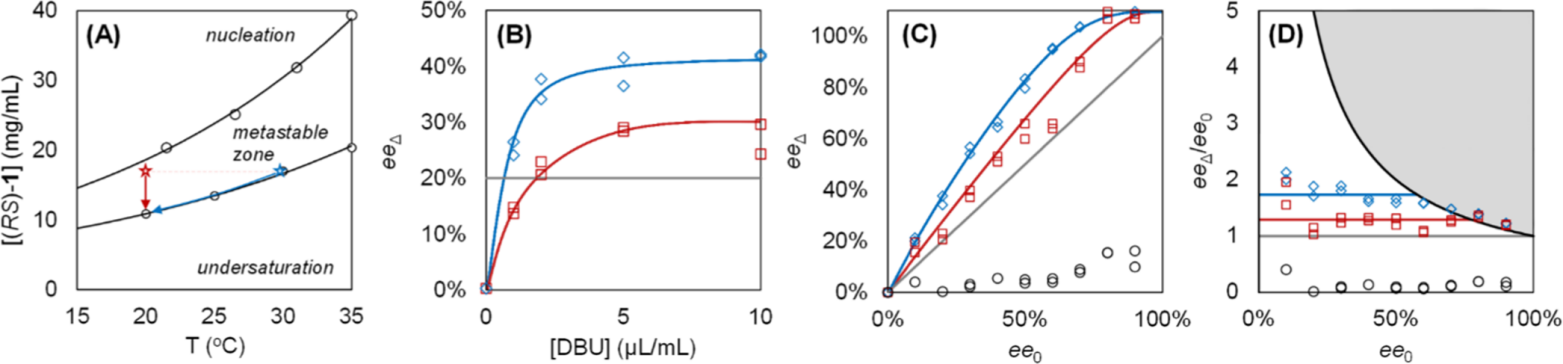
Erosion, consolidation, and amplification of solid-phase ee of **1** during crystal growth depends on the balance between the crystallization and racemization rate. (A) Temperature-dependent phase diagram with the metastable zone of (*RS*)-**1** in MeCN. The stars indicate the seeding conditions: abrupt (red, seed at 20 °C) or slow (blue, seed at 30 °C) cooling. (B, C) Enantiomeric excess of crystallized material ee_Δ_ is shown as a function of (B) the amount of racemization catalyst DBU (ee_0_ = 20%) and (C) as a function of the enantiomeric excess of the seed crystals ee_0_ ([DBU] = 2 μL/mL) for abrupt cooling (red squares), slow cooling (blue diamonds), and no racemization (black circles, [DBU] = 0). (D) Experimental amplification factor ee_Δ_/ee_0_ as a function of ee_0_ showing approximately constant amplification up to the theoretical limit 100%/ee_0_ (gray zone). Blue and red lines (B–D) are guides to the eye.

To determine the experimental parameters for crystal growth, we establish the temperature-dependent solubility of (*RS*)-**1** (Figure 2A) and determine the supersaturation at which spontaneous nucleation occurs to define the metastable zone wherein merely growth takes place (Figure 2A). The region between 20 and 30 °C in the phase diagram is within the metastable zone and suitable to grow substantial amounts of material. To grow in this region of the phase diagram, we first saturate a solution at 30 °C in the presence of various amounts of racemization catalyst (0, 1, 2, 5, or 10 μL/mL DBU). We then abruptly cool the solution down to 20 °C to create a clear supersaturated solution, which is then added to a solid phase of enriched seed crystals (25 mg/mL of 20% ee_0_ in (*R*)-**1**) to initiate growth (Figure 2A). The resulting slurry is kept homogeneous using a shaking platform at the lowest possible setting (ca. 300 rpm). We use shaking—rather than stirring—to focus on crystal growth effects while avoiding undesired attrition and secondary nucleation. After 90 min, we determine the enantiomeric composition of the solid phase using chiral HPLC. To analyze the change of the solid-phase composition as a result of growth, we calculate the enantiomeric excess of the grown material (ee_Δ_) using eq 1
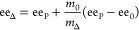
1where ee_P_ is the ee after growth, *m*_0_ is the mass of seed crystals, ee_0_ is the ee of the seed crystals, and *m*_Δ_ is the mass of grown material as determined by the difference in solubility between 20 and 30 °C.

After seeding, the phase diagram predicts that *m*_Δ_ = 6 mg/mL of **1** is grown on the seeds, i.e., the solid-phase concentration increases to 31 mg/mL. We thus expect the average size of crystals to increase, which is confirmed by comparing scanning electron microscopy images of the crystals before and after growth (data shown in Section H of the Supporting Information). In the absence of racemization (0 μL/mL DBU), we observe erosion of the solid-phase enantiopurity from ee_0_ = 20% to ee_P_ = 16%. This ee_P_ corresponds to the precipitation of equal amounts of *R* and *S* (3 mg/mL of (*R*)-**1** and 3 mg/mL of (*S*)-**1**), yielding ee_Δ_ = 0 from eq 1 (Figure 2B). Consistent with scenario (i) (Figure 1), in the absence of a racemization catalyst, disproportional growth of minority solid-phase (*S*)-**1** thus results in erosion of the solid-phase enantiopurity.

In the presence of 1 μL/mL racemization catalyst, however, we find that unequal amounts of (*R*)-**1** and (*S*)-**1** have precipitated. The grown material is slightly enriched in (*R*)-**1** with ee_Δ_ = 15% (Figure 2B). Increasing the catalyst concentration further to 2 μL/mL DBU results in growing even more enriched material with ee_Δ_ ≈ 20% = ee_0_, meaning no erosion of the solid-phase enantiopurity has occurred at all during growth (Figure 2B). As described by scenario (ii) of our mechanistic framework (Figure 1), under these relatively slow and limiting racemization conditions, both crystal populations grow proportionally to their concentration, thereby consolidating the solid-phase enantiopurity.

Strikingly, for high concentrations of the racemization catalyst (5 and 10 μL/mL DBU, i.e., fast racemization compared to crystallization), the solid phase enriches beyond the initial ee during growth, with ee_Δ_ = 30% > ee_0_, thus demonstrating amplification of the solid-phase ee (Figure 2B). This asymmetric amplification implies that the majority crystal population (*R*)-**1** grows faster than the minority population (*S*)-**1**. Consistent with scenario (iii) (Figure 1), the faster growth rate of (*R*)-**1** crystals results in a depletion of this enantiomer in the liquid phase, which is then replenished by the conversion of (*S*)-**1** to (*R*)-**1** through racemization. Since we only observe this enantioenrichment for high concentrations of the racemization catalyst, these results indicate that only high racemization rates allow for sufficiently fast conversion of the minor enantiomer to the major enantiomer to compensate for the disbalance in growth rates between the two crystal populations. For 10 μL/mL DBU, no further enrichment is observed over 5 μL/mL DBU (Figure 2B), demonstrating that there is a limit to the enrichment that can be achieved by increasing the racemization rate, as the rate of crystal growth cannot keep up with the rate of racemization. Increasing the racemization rate relative to the crystallization rate thus determines whether erosion, consolidation, or amplification occurs during crystal growth.

Our framework suggests that tuning the crystallization rate compared to the racemization rate also enables control over the degree of solid-phase enrichment. Amplification is expected when racemization is fast compared to crystallization, prompting us to slow down the crystallization rate. To this aim, we seed a saturated solution and subsequently slowly increase the supersaturation by slow cooling (Figure 2A) instead of seeding a cooled supersaturated solution directly (as in the previous abrupt cooling experiment). Specifically, we decrease the crystallization rate by slow linear cooling of the saturated solution from 30 to 20 °C in 90 min (0.11 °C/min) in the presence of the seed crystals (20% ee_0_ in (*R*)-**1**) for the previously used racemization catalyst concentrations (0, 1, 2, 5, or 10 μL/mL DBU). We observe that slowing down the crystallization rate yields a systematic increase of the enrichment in the grown material compared to fast growth (Figure 2B). Hence, maximizing the ratio of the racemization rate to crystallization rate results in amplification of the initial solid-phase enantiomeric excess during crystal growth.

We investigate how the amplification during growth is governed by the proportions of the initial crystal populations ee_0_. For a fixed racemization catalyst amount (0 and 2 μL/mL DBU), we vary the initial enantiomeric excess of the seed crystals (10–90%) for both abrupt and slow cooling experiments (Figure 2A) and plot the enrichment of the grown material ee_Δ_ as a function of the initial solid-phase enrichment ee_0_ (Figure 2C). In the absence of racemization (0 μL/mL DBU), we find ee_Δ_ ≈ 0 for all initial ee’s, which is consistent with equal precipitation of both enantiomers as calculated from the phase diagram. In the presence of racemization (2 μL/mL DBU) and abrupt cooling (seeding at 20 °C), the initial ee is preserved during growth (ee_Δ_ ≈ ee_0_) for all enantiomeric excesses of the seed. With racemization and slow cooling (seeding at 30 °C, linear cooling to 20 °C with 0.11 °C/min), we observe amplification for all initial ee’s (ee_Δ_ > ee_0_). In agreement with our framework (Figure 1), increasing the racemization rate compared to the crystallization rate thus results in increased asymmetric crystal growth for all initial proportions of the crystal populations.

To quantify the extent of amplification, we define the experimental amplification factor ee_Δ_/ee_0_: a relative measure for the enrichment in the grown material compared to the enrichment of the initial seed grown onto. Because ee_Δ_ is theoretically limited to 100%, the amplification factor is in turn limited to 100%/ee_0_ and thereby constrains ee_Δ_/ee_0_ for large ee_0_. We plot ee_Δ_/ee_0_ as a function of ee_0_ for all experiments (Figure 2D). In the absence of DBU, ee_Δ_/ee_0_ is indeed equal to 0. In the presence of 2 μL/mL DBU, we find amplification factors of ee_Δ_/ee_0_ ≈ 1.3 > 1 (abrupt cooling) and ee_Δ_/ee_0_ ≈ 1.7 > 1 (slow cooling), corresponding to the enrichment of the solid phase beyond its initial enantiomeric excess. Moreover, ee_Δ_/ee_0_ remains approximately constant for all ee_0_ up to the theoretical limit, indicating that both small and large enantiomeric excesses in seeds can be amplified equally well.

**Figure 3:**
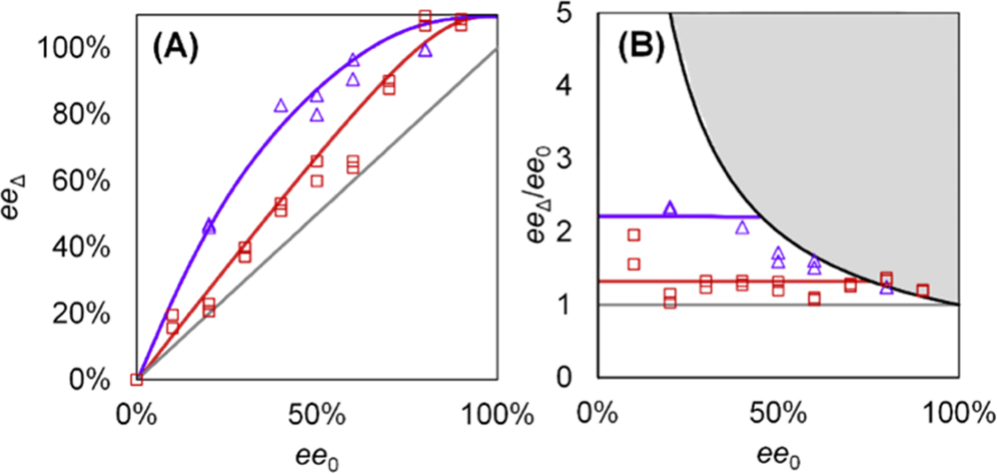
Amount of seed crystals controls the level of amplification of the enantiomeric excess. Decreasing *m*_0_ from 25 mg/mL (red squares) to 0.5 mg/mL (purple triangles) results in (A) higher enantiopurity of grown material ee_Δ_ and (B) increase of the amplification factor ee_Δ_/ee_0._ Purple and red lines are guides to the eye.

We realize that—besides the rates of crystallization and racemization—we can further tune the amplification factor by controlling the mass of grown material (*m*_Δ_) relative to the mass of seed crystals (*m*_0_). A smaller *m*_Δ_/*m*_0_ results in less crystal growth and therefore less amplification of the enantiomeric excess. In contrast, a larger *m*_Δ_/*m*_0_ results in more crystal growth and therefore a higher degree of amplification: consider using the product of a first crystallization experiment as a seed for a subsequent experiment. Such a recursive procedure would expectedly lead to a net increased and compounded amplification factor. One effective way for increasing *m*_Δ_/*m*_0_ is by decreasing the amount of seed crystals. To demonstrate this, we repeat the abrupt cooling experiment with 2 μL/mL DBU but using 50 times smaller amount of seed crystals (*m*_0_ = 0.5 instead of 25 mg/mL) (Figure 3). We indeed find an increase in the amplification factor from 1.3 (*m*_0_ = 25 mg/mL) to 2.2 (*m*_0_ = 0.5 mg/mL) (Figure 3B). In fact, seeds of only 60% ee already result in virtually exclusive growth of the majority phase (ee_Δ_ > 90%) (Figure 3A). Hence, prolonged growth onto a seed with a small enantiomeric excess can lead to large chiral amplification and yield a highly enantiopure product.

The mechanistic framework describing the observed amplification during growth of **1** is—to a large extent—compound-independent, suggesting that these principles readily extend to other racemizable conglomerates. To show the generality of these chiral amplification phenomena, we perform a demonstration of amplification during growth for *tert*-leucine precursor **2** (3,3-dimethyl-2-((naphthalen-2-ylmethylene)amino)butanenitrile) (Figure 1C). Enantiopure *tert*-leucine is widely used in the synthesis of modified peptides and antiviral agents such as the HIV protease inhibitor atazanavir.^60,61^ Compound **2** crystallizes as a conglomerate and also readily undergoes racemization in the presence of DBU in methanol.^40,49,62^ To demonstrate that the chiral amplification is also independent of the racemization catalyst, we use the organic base 1,1,3,3-tetramethylguanidine (TMG), which we show racemizes **2** as well (Figure S1). As proof of generality, akin to the abrupt cooling experiments for **1**, we create a supersaturated solution of (*RS*)-**2** (30 mg/mL) in the presence of 100 μL/mL TMG at 20 °C. After seeding with 0.5 mg/mL of seed crystals with ee_0_ = 60%, we obtain a solid phase with ee_P_ = 92% and ee_Δ_ = 97% (*m*_Δ_ = 3 mg/mL). The resulting amplification factor ee_Δ_/ee_0_ = 1.66 is close to the theoretical limit (100%/ee_0_ = 1.70), demonstrating that large asymmetric amplification during crystal growth can be achieved with different choices of conglomerate, solvent, and racemization catalyst.

Although the exact nature of the asymmetric crystal growth is still unclear, we anticipate that this mechanism could also play a decisive role during deracemization processes in which not only growth but also dissolution take place. To explore these potential mechanistic analogies, we implemented the chiral amplification factor in a simple analytical model to describe both the kinetics of attrition-enhanced as well as temperature cycling-induced deracemization (see Section I of the Supporting Information for details and the derivations). We assume that chiral amplification occurs during growth as described by a constant amplification factor ee_Δ_/ee_0_. We further assume that dissolved material is racemic such that ee_Δ_ = 0 during dissolution steps. Combining these assumptions, we can describe the evolution of the ee for consecutive growth and dissolution steps by eq 2
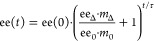
2where we introduce a typical cycle time τ. To describe attrition-enhanced deracemization, where *m*_Δ_ and τ are less well defined, eq 2 can be shown to simplify to the classical exponential description of eq 3
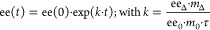
3

We find that previously reported kinetic data from both temperature cycling-induced and attrition-enhanced deracemization experiments are successfully described by eqs 2 and 3 (fits included in Section I of the Supporting Information).^40,59^ Moreover, trends for the deracemization rate *k* predicted by our model agree with those reported in the literature from experiments.^28,40,63^ This analysis shows that the introduction of an amplification factor to describe asymmetric crystal growth is sufficient to account for the kinetics of both deracemization methods. We therefore submit that a general mechanism involving chiral amplification during crystal growth may play an essential role in all of these crystallization-induced deracemization methods.

## Summary and Outlook

In summary, guided by a mechanistic framework, we find that during crystal growth, the interplay between racemization and crystallization rates can result in either erosion, consolidation, or amplification of the enantiomeric excess of the seed crystals. The faster growth of the majority population of enantiomorphic seed crystals is at the core of this remarkable chiral amplification mechanism and can lead to large chiral amplification, even for seed crystals with a small enantiomeric excess. Surprisingly, solution-phase racemization always leads to growing material with enantioenrichment (ee_Δ_ > 0), regardless of the conditions under which growth is performed, and thus prevents the erosion observed without racemization.

The here-observed imbalance in crystallization rates reveals an intriguing form of asymmetric crystal growth that challenges our current understanding. The kinetics of temperature cycling-induced and attrition-enhanced deracemization as well as the underlying nonlinear effects presented here appear to be well described by the introduced experimental amplification factor. Our results thus suggest that asymmetric crystal growth effects play an essential part in driving deracemization processes of which crystal growth is a major component and can advance our understanding of the surprising amplification effects driving these deracemization processes.^25,27,28,30−32,35,41,45,52,64^ Moreover, this form of asymmetric crystal growth might also explain why an enantiopure compound can grow at the expense of a stable racemic compound.^65^ Nevertheless, the molecular mechanism remains elusive and could involve processes at the crystal—liquid interface and nonclassical crystallization mechanisms such as enantiomer-specific oriented attachment (see Section H of the Supporting Information).^31,66^ The next steps are aimed at further clarifying the roles of crystal size effects and the quantitative relationship between crystal growth and racemization rates to ultimately unravel the molecular mechanism that lies at the core of this remarkable form of asymmetric crystal growth.

Our results also hold direct relevance for the practical production of enantiopure building blocks as they provide a tangible basis for the optimization of crystallization-based deracemization processes. We challenge the general consensus that large amounts of seed crystals with the highest enantiopurity are essential, which stems from the tacit understanding that the purity of the seeds determines the maximum achievable enantiomeric excess of the final product.^2,53−56^ Counterintuitively, we show that the level of enantioenrichment can even be increased by decreasing the amount of seed crystals and that amplification of ee can even be achieved for small amounts of seed crystals of low enantiopurity. Specifically, we realize that industrial crystallizers are ideal to achieve the desired conditions for large enantioenrichment: small amounts of seed crystals, large amounts of grown material, and slow rates of crystallization combined with fast solution-phase racemization, hence outlining the potential for practical production of chiral molecules.

More broadly, the here-introduced asymmetric crystal growth process may hold relevance for understanding the emergence and further amplification of enantiopure building blocks in the origin of life scenarios. Specifically, we envision that asymmetric crystal growth-induced amplification of enantiomeric excesses can play an essential role in amplifying minute chiral imbalances that are generated during spontaneous symmetry-breaking processes, such as crystal nucleation from clear solutions, as described by Havinga and Kondepudi.^20−23,58^ In conclusion, the here-introduced chiral amplification process and mechanistic framework can guide the practical production of biologically active enantiopure molecules and fit well in scenarios for the origin of single chirality in nature starting from (racemizable) conglomerates.

## Supporting Information Available

Experimental section, methodological details: HPLC analysis, seed crystal preparation, temperature-dependent solubility, metastable zone determination, racemization of compound **2**, micrographs of crystals before and after growth, and kinetic model based on the amplification factor (PDF)
