# Supplementary material for: Chiral Amplification through the Interplay of Racemizing Conditions and Asymmetric Crystal Growth
Source: J Am Chem Soc. 2022 Dec 19;145(1):436–42. doi: 10.1021/jacs.2c10584 (PMC9837840; doi:10.1021/jacs.2c10584)
Supplement: Supplementary file 1 — ja2c10584_si_001.pdf [file ja2c10584_si_001.pdf]

Supporting Information

Chiral Amplification through the Interplay of Racemizing Conditions and Asymmetric Crystal Growth

Sjoerd W. van Dongen[a], Imane Ahlal[a], Michel Leeman[b], Bernard Kaptein[c], Richard M. Kellogg[b], Iaroslav Baglai[a]*, and Willem L. Noorduin[a,d]*

[a] AMOLF, Science Park 104, 1098 XG, Amsterdam, The Netherlands.

[b] Symeres, Kadijk 3, 9747 AT, Groningen, The Netherlands.

[c] InnoSyn BV, Urmonderbaan 22, 6167 RD, Geleen, The Netherlands.

[d] Van 't Hoff Institute for Molecular Sciences, University of Amsterdam, Science Park 904, 1098 XH, Amsterdam, The Netherlands.

* Email: iaroslav.baglai@gmail.com; noorduin@amolf.nl

Table of Contents

	Supplementary Figure	p. S-1
	Experimental Details	p. S-2
A.	General remarks	p. S-2
B.	HPLC analysis	p. S-2
C.	Seed crystal preparation	p. S-3
D.	Temperature dependent solubility and metastable zone of **1**	p. S-4
E.	Growth experiments for compound **1**	p. S-5
F.	Racemization of compound **2**	p. S-7
G.	Growth experiments for compound **2**	p. S-8
H.	Comparison of crystals before and after growth	p. S-9
I.	Kinetic model based on the amplification factor	p. S-10

Supplementary Figure

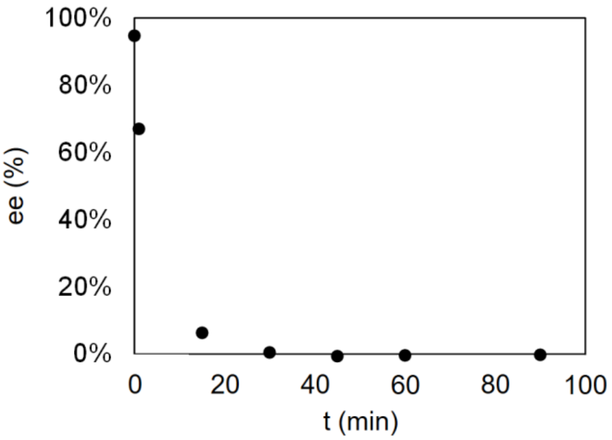

Figure S-1. Racemization kinetics for compound **2** using TMG, starting from a virtually enantiopure solution (t=0: 0.5 mg/mL (R)-**2** and 1 µL/mL TMG in MeOH).

Experimental Details

A. General remarks

Chiral HPLC analyses were performed using an Agilent Technologies Infinity 1260 HPLC sysem. The thermostat used was manufactured by Huber (Huber Heat Regulator RS2232, range: 5 – 60 °C). All solvents used (n-heptane, 1-propanol, acetonitrile and methanol) were HPLC grade (≥ 99%) and obtained from VWR chemicals. For sample preparation and analysis, 0.2 µm PTFE syringe filter (VWR Internationals) and 2 mL HPLC vials (29651-U Supelco, Merck) were used. To be able to experiment with multiple solutions at the time, a block from aluminium with milled holes for vials was fabricated in-house by the AMOLF Precision Manufacturing Department (suitable for either 2 mL or 7 mL vials). When shaking, a VWR Standard vortex mixer was used. When stirring, 6x3mm cylindrical PTFE stirring bars (VWR) combined with a standard VWR hotplate were used. The 7 mL vials used in experiments were sourced from Merck (27150-U Supelco). The racemization catalysts used are 1,8-Diazabicyclo[5.4.0]undec-7-ene (Across Organics) and 1,1,3,3-tetramethylguanidine (Sigma-Aldrich). The compounds **1** and **2** used were obtained over the course of previous research.[47,49]

B. HPLC analysis

<u>Sample preparation</u>

For liquid phase samples: 50 – 100 µL of liquid was mixed with 1 mL of IPA by vortex.

For solid phase samples: 0.5 – 1.0 mg of solid material was dissolved in 1.5 mL of IPA by vortex and subsequent ultrasonication (10 minutes).

<u>HPLC method</u>

HPLC analysis was performed on a chiral column (CHIRALPAK IA (250 x 4.6 mm, 5µm)) with a mobile phase consisting of n-heptane and 1-propanol. For compound **1** (Figure S-2), the eluent is mixed in a 7:3 ratio (heptane:IPA). For compound **2** (Figure S-3), the eluent ratio is 95:5 (heptane:IPA). The flow rate was 0.7 mL/min, injection volume 4 µL, and detection was performed by UV-detector (wavelength: 220 nm). Each run had a total time of 10 to 12 minutes.

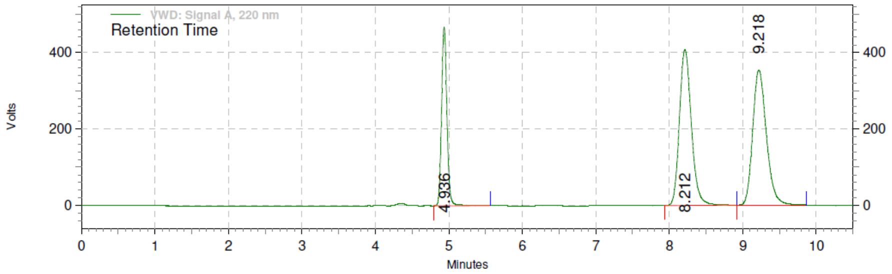

Figure S-2. Typical chromatogram with retention times for **1**: 4.94 min for anisole, 8.12 min for (R)-**1**, and 9.12 min for (S)-**1**.

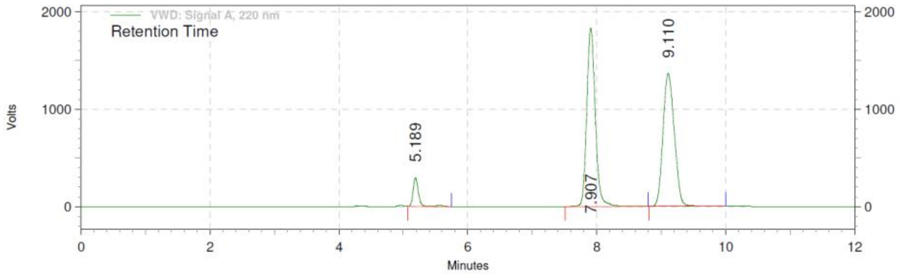

Figure S-3. Typical chromatogram with retention times for **2**: 5.19 min for anisole, 7.91 min for (S)-**2**, and 9.11 min for (R)-**2**.

Concentration determination
All experiments were run in MeCN or MeOH with an added 0.2 wt% anisole. The anisole acts as internal standard. By constructing calibration curves, the concentration of (R) and (S) in each liquid phase sample could be determined (Figure S-4).

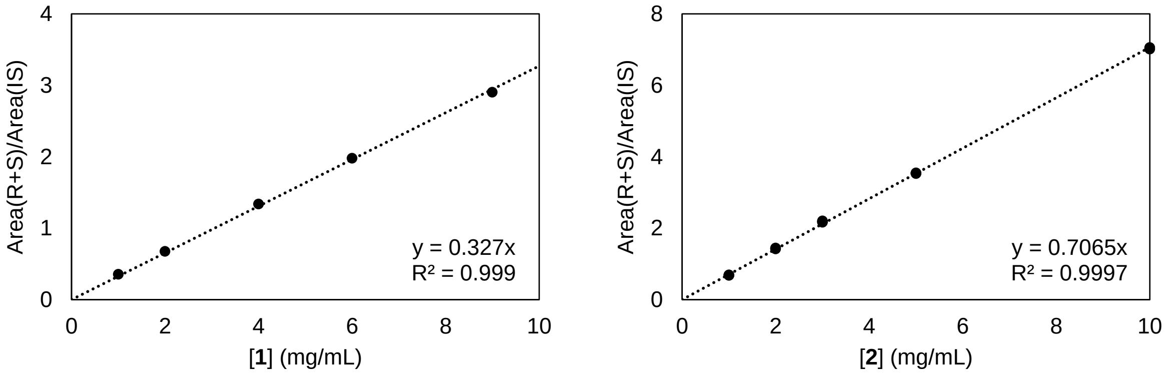

Figure S-4. Calibration curves for **1** (left, in MeCN) and **2** (right, in MeOH) with 0.2wt% anisole.

C. Seed crystal preparation

Seed crystals of a specific enantiomeric excess (e.e.) were prepared from racemic and enantiopure source crystals as follows. First, the amount of required racemic (m_{RS}) and enantiopure crystals (m_R) was calculated using the following formulae (m_{R+RS} is the total required seed weight):

$$m_R = \mathrm{ee} \cdot m_{R+RS}$$
$$m_{RS} = (1 - \mathrm{ee}) \cdot m_{R+RS}$$

These amounts of racemic and enantiopure crystals were then weighed and combined in a mortar and mixed with a spatula. Following the initial mixing, the powder was vigorously mixed using the pestle to form a homogeneous powder. By taking and analysing various samples of the resulting powder, the seed crystal enantiomeric excess was validated by HPLC.

The following seeds were prepared for **1**: 10%, 20%, 30%, 40%, 50%, 60%, 70%, 80% and 90% ee in R. The following seed was prepared for **2**: 60% ee in R.

To assess the size and physical characteristics of the seed crystals, SEM micrographs were taken of all batches (FEI Verios 460; 6nm Cr). Seed crystals were, on average, between 5 and 50 μm in size (Figure S-5). The seeds were reproducible and consistent between batches.

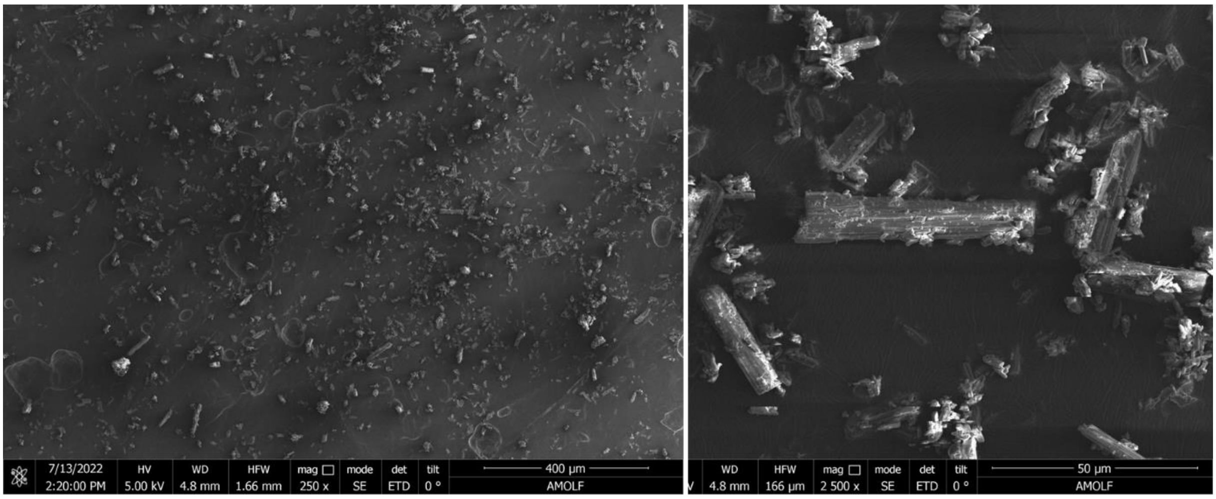

Figure S-5. Representative SEM micrographs of seed crystals at lower (left) and higher (right) magnification. Example micrographs displayed originate from seeds of 20% ee in R for compound **1**.

D. Temperature dependent solubility and metastable zone of 1

Temperature dependent solubility

The temperature dependent solubility of **1** was determined by heating a slurry of (RS)-**1** in MeCN (with 0.2 wt% anisole) to the desired temperature (20, 25, 30, 35 °C) in a 7 mL vial under magnetic stirring. After three hours, a liquid phase sample was taken and submitted for HPLC analysis. The resulting temperature solubility data are given in Figure 2(a) (*main text*).

Furthermore, we validated that the solubility is independent of the concentration of racemization catalyst (DBU) at both 20 and 30 °C (Figure SI-6).

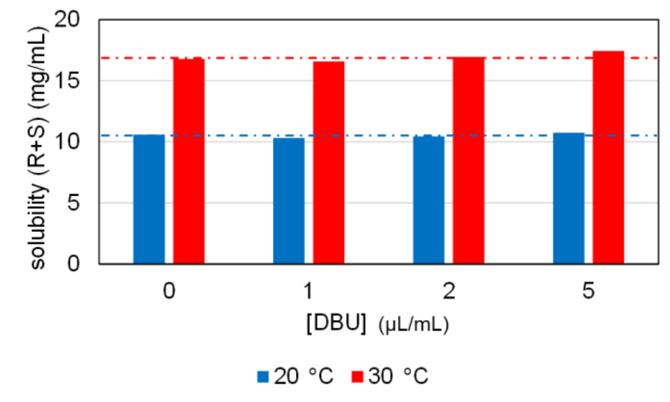

Figure S-6. Solubility of (R,S)-**1** at 20 °C (left-side blue bars) and 30 °C (right-side red bars) for various concentrations of DBU. The dotted lines indicate the level for no (zero) amount of catalyst.

Metastable zone determination

To probe the metastable zone of **1**, saturated solutions were prepared at temperatures of 35, 40, 45 and 50 °C in 20 mL vials (10 mL solution; the smaller volumes used in growth experiments would correspond to even wider metastable zones than those measured here). These solutions were placed in a pre-heated water bath and the thermostat was switched off. Under mild magnetic stirring, the temperature of the solution was monitored while it slowly lowered towards room temperature (20 °C). As soon as nucleation was observed, the temperature was noted, corresponding to the critical supersaturation for that initial concentration. The resulting metastable zone is displayed in Figure 2(a) of the main text.

Independently, we verified that a solution saturated at 30 °C remains clear of nucleation for over 2 weeks at 20 °C (without stirring), after which single crystals slowly emerge.

To assess the validity of the metastable zone determination and the dependability on racemization catalyst (DBU) concentration, we checked the nucleation time for 1 mL (in 2 mL HPLC vials) and 5 mL (in 7 mL vials) solutions (identical preparation as above) with varying concentrations of DBU (Table S-1) under stirring. No nucleation was observed during the experimental timescales (max. 3 hours) and the MSZ width appears DBU-independent.

Table S-1. Metastable zone validation for compound **1** under experimental conditions. Cross (x) indicates that nucleation was observed. Supersaturated solution prepared at 30 °C, cooled to 20 °C.

V (mL)	[DBU] (μL/mL)	1.5 hr	3.0 hr	24 hr
1	2			
1	5			x
1	10			
5	2			x
5	5			x
5	10			

E. Growth experiments for compound 1

<u>Preliminaries</u>

Unless stated otherwise, growth experiments were carried out in 2 mL vials, placed in a custom-made multi-vial holder which was thermoregulated to 20 °C by a Huber thermostat (setting 19.8 °C). The multi-vial holder was placed on top of a shaker and shaken throughout the entire experiment at medium velocity (ca. 300 rpm).

Generally, for each growth experiment, 25 mg of seed crystals were weighed into a 2 mL vial. A supersaturated solution was prepared by mixing 200 mg (RS)-**1** with 5 mL internal standard solution in a 7 mL vial equipped with a magnetic stirring rod at 30 °C. In case of experiments under racemizing conditions, DBU was added as well. The mixture was stirred for 30 to 60 minutes. Then, the stirring was stopped to allow the suspension to sediment and the clear supernatant was filtered (2 µm PTFE syringe filter). The filtrate was cooled back to 20 °C over the course of 15 minutes and used as 'supersaturated solution' directly afterwards.

Note: Whenever vortexing suspensions, make sure to lower the speed gently as to avoid crystals sticking to the vial walls.

<u>Reference experiments (without racemization)</u>

The vial with the seed crystals was placed in the vial holder. The reference experiments (not under racemizing conditions) were initiated by adding 1 mL of supersaturated solution to the vial containing the seed crystals under shaking (T = 20 °C). The vial was then closed, swiftly vortexed, and placed back in the multi-vial holder. After 90 to 120 minutes, the liquid phase was sampled by filtering 125 µL through a 2 µm PTFE syringe filter and the filtrate was analysed further as the 'liquid phase'. Then, the remaining suspension was cast on top of filter paper laid down on glass filter connected to a vacuum filtration set-up (whilst under active vacuum). Two separate samples of the solids were taken using a Pasteur pipette analysed further as the 'solid phase' (Figure S-7).

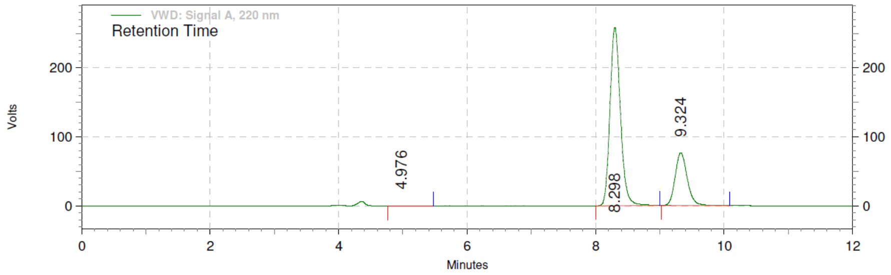

VWD: Signal A, 220 nm Results

Retention Time	Area	Area %	Height	Height %
4.976	32227	0.05	1903	0.03
8.298	48892148	74.48	4332637	77.11
9.324	16716244	25.47	1284520	22.86

Figure S-7. Typical solid phase chromatogram following a reference experiment (ee_0 = 60%).

<u>Growth under racemization conditions</u>

The vial with the seed crystals was placed in the vial holder. The seminal experiments under racemizing conditions were then initiated by adding 1 mL of supersaturated solution (now also containing DBU) to the vial containing the seed crystals under shaking (T = 20 °C). The vial was then closed, swiftly vortexed, and placed back in the multi-vial holder. After 90 to 120 minutes, sampling proceeded identically to the procedure stated above under 'reference experiments' (Figure S-8).

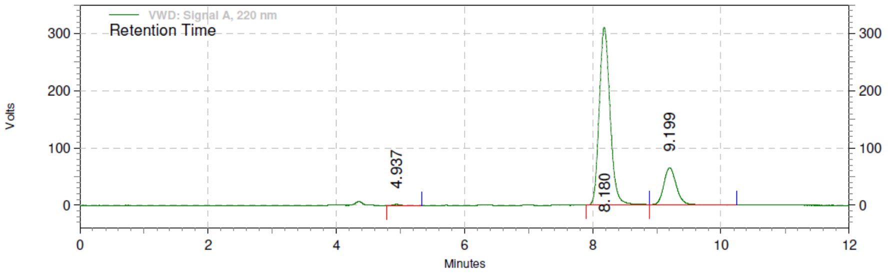

VWD: Signal A, 220 nm Results

Retention Time	Area	Area %	Height	Height %
4.937	150362	0.20	25888	0.41
8.180	61282845	79.94	5219307	82.26
9.199	15226482	19.86	1099570	17.33

Figure S-8. Typical solid phase chromatogram following an abrupt growth experiment ([DBU] = 2 µL/mL, ee_0 = 60%).

<u>Slow growth</u>

The thermostat of the multi-vial holder was set to 31 °C (actual temperature 30 °C). The vial with the seed crystals was placed in the vial holder and equilibrated for 30 minutes, under continuous shaking. The experiments (under racemizing conditions) were initiated by adding 1 mL of supersaturated solution (containing the DBU) to the vial containing the seed crystals under shaking. The vial was then closed, swiftly vortexed, and placed back in the multi-vial holder. A cooling ramp was started, in which the thermostat temperature was linearly decreased to 20 °C over the course of 90 minutes as to allow for gradual growth. The system remained at 20 °C for 30 more minutes. Sampling proceeded after approximately 120 minutes (counted from the mixing between seeds and solution), identically to the procedure stated above under 'reference experiments' (Figure S-9).

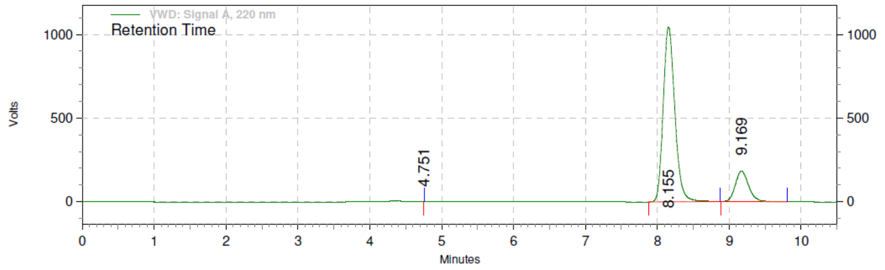

VWD: Signal A, 220 nm Results

Retention Time	Area	Area %	Height	Height %
4.751	105	0.00	196	0.00
8.155	197431306	83.42	17574501	85.20
9.169	39231656	16.58	3053171	14.80

Figure S-9. Typical solid phase chromatogram following a slow growth experiment ([DBU] = 2 µL/mL, ee_0 = 60%).

<u>Growth kinetics</u>

Samples after growth are taken after 90 to 120 minutes. To verify that growth has indeed completed after this period of time and the composition of the solid phase does not change afterwards, the experiment under 'Growth under racemization conditions' was performed for seeds with 20% ee in R and sampled after 90 and 200 minutes. Both solid and liquid phase samples had identical concentration and ee, respectively, indicating that growth is completed after 90 minutes and sampling can occur at any time afterwards.

Prolonged growth of small seed crystal amounts

For the experiments in which small amounts of seed crystals are used to enable prolonged growth, the following altered procedure was followed, based on that for abrupt growth ("Growth under racemization conditions"). A 7 mL vial containing a stirring rod was now placed in the vial holder on a stirring plate (T = 20 °C). Identical to previous experiments, a supersaturated solution was prepared in the presence of DBU. After adding 2 mL of supersaturated solution to the 7 mL vial, 1 mg of seed crystals were added as well under stirring conditions. The vial was then quickly vortexed, swiftly closed and placed back in the vial holder. Mild stirring continued throughout the experiment. Sampling proceeded after approximately 3 hours, counted from the mixing between seeds and the supersaturated solution, identically to the procedure stated above under 'reference experiments' (Figure S-10).

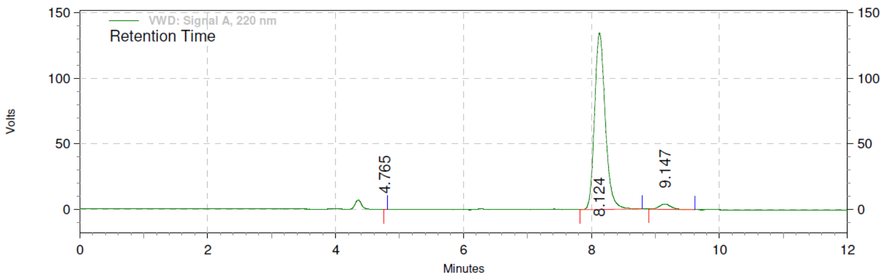

VWD: Signal A, 220 nm Results

Retention Time	Area	Area %	Height	Height %
4.765	763	0.00	439	0.02
8.124	26350299	96.72	2262468	97.02
9.147	893759	3.28	68954	2.96

Figure S-10. Typical solid phase chromatogram following the use of a small amount of seed crystals for prolonged growth ([DBU] = 2 µL/mL, ee_0 = 60%).

F. Racemization of compound 2

To verify the racemization of compound **2** using the organic base TMG, a solution of (R)-**2** was prepared in MeOH (0.5 mg/mL) in the presence of 0.2wt% anisole in a 2 mL HPLC vial. The vial was sampled using the HPLC directly (t = 0, Figure S-11). Then, 1 µL/mL TMG was added and sampling was performed at regular time intervals (1, 15, 30, 45, 60, 90 minutes) directly by the HPLC. After 30 minutes, racemization was complete (Figure S-11). The full racemization kinetics are displayed in Figure S-1.

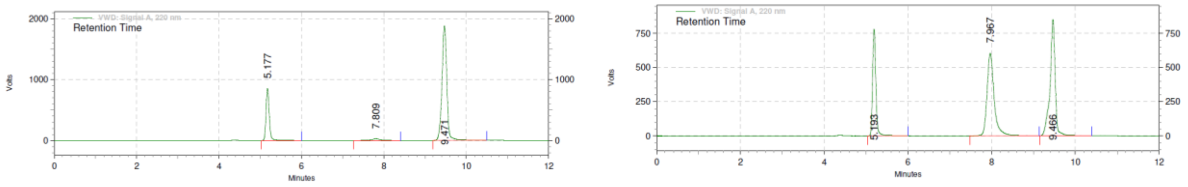

VWD: Signal A, 220 nm Results

Retention Time	Area	Area %	Height	Height %
5.177	78149149	22.72	14307525	30.86
7.809	6946303	2.02	449308	0.97
9.471	258801222	75.26	31599652	68.17

VWD: Signal A, 220 nm Results

Retention Time	Area	Area %	Height	Height %
5.193	71472543	22.83	13099190	34.91
7.967	120275073	38.41	10152153	27.06
9.466	121357044	38.76	14270691	38.03

Figure S-11. Chromatograms of the solution at t = 0 (virtually enantiopure in R-**2**, left) and t = 30 minutes (virtually racemic **2**, right) of racemization with TMG (t=0: 0.5 mg/mL (R)-**2** and 1 µL/mL TMG in MeOH).

G. Growth experiments for compound 2

Temperature dependent solubility

The temperature dependent solubility of **2** was determined by heating a slurry of (RS)-**2** in MeOH (with 0.2 wt% anisole and 100 µL/mL TMG) to the desired temperature (20, 25, 30 °C) in a 7 mL vial under magnetic stirring. After three hours, a liquid phase sample was taken and submitted for HPLC analysis. The resulting data are given in Figure S-12.

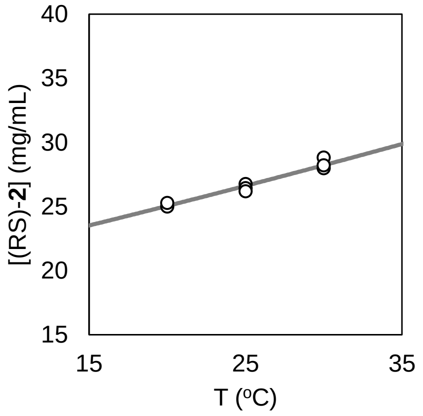

Figure S-12. Temperature dependent solubility for **2** (in MeOH) in the presence of 100 µL/mL TMG.

Metastable zone

To validate that growth experiments take place within the metastable zone of **2**, it was visually verified that a solution saturated at 40 °C with [(RS)-**2**] = 30.4 mg/mL in MeOH, containing 100 uL/mL TMG and 0.2 wt% anisole, remains clear of nucleation for at least a day after having been cooled to 20 ºC (no stirring). The solubility difference (30.4 – 25.1 = 5.3 mg/mL) is similar to that imposed for compound **1**.

Growth experiment

A solution of (RS)-**2** was prepared in MeOH at 40 °C (30.4 mg/mL) in the presence of 100 µL/mL TMG and 0.2 wt% anisole. The solution was cooled down to 20 °C. Similar to the experiment described under "Prolonged growth of small seed crystal amounts" for compound **1**, in a 7 mL vial, 1 mg of seed crystals (60% ee enriched in R) was added to 2 mL of supersaturated solution under the mildest of stirring conditions. After 3 hours, both solid and liquid phase were sampled (Figure S-13).

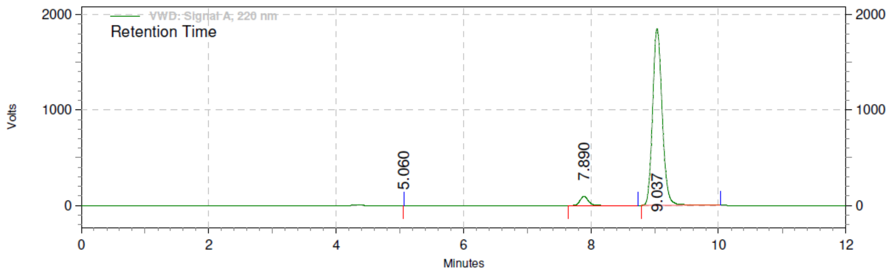

VWD: Signal A, 220 nm Results

Retention Time	Area	Area %	Height	Height %
5.060	95	0.00	267	0.00
7.890	13877683	4.10	1561720	4.78
9.037	324260793	95.90	31081405	95.21
Totals	338138571	100.00	32643392	100.00

Figure S-13. Solid phase chromatogram following the growth experiment for compound **2** ([TMG] = 100 µL/mL, ee_0 = 60%).

H. Comparison of crystals before and after growth

Seed crystals of 20% ee (25 mg/mL) in *R* of compound **1** were subjected to the growth conditions listed under ‘Growth under racemization conditions’ in the presence of 2 µL/mL DBU. Before and after growth, solid phase samples were deposited on carbon stickers adhered to 1 cm^2 aluminium substrates by a sprinkling method. Excess crystals, that did not adhere to the sticker, were removed using N_2 flow. The samples were then coated with a 11.3 nm layer of Chrome and directly imaged on a FEI Verios 460 Scanning Electron Microscope in immersion mode.

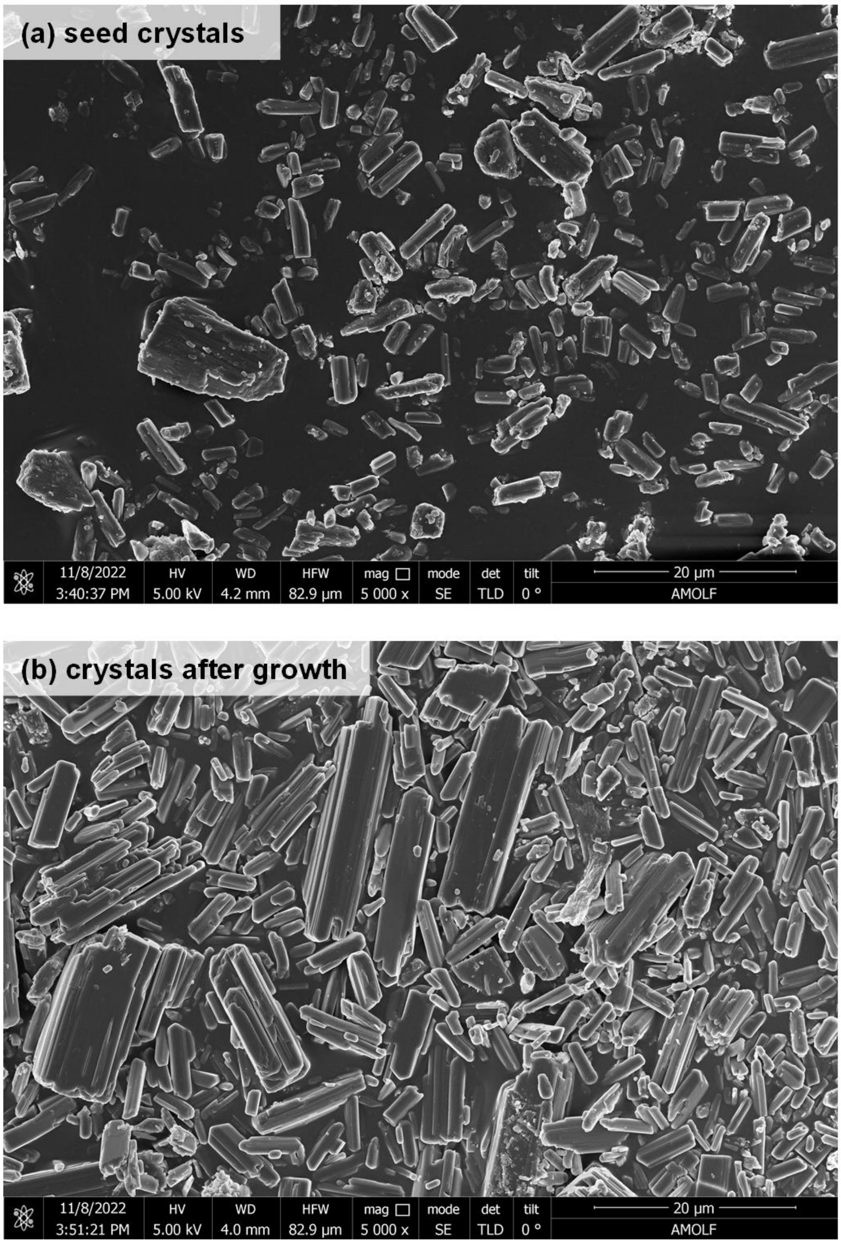

Figure S-14. Scanning electron micrographs (SEM) of crystals of compound **1** (a, top) before (20% ee in R) and (b, bottom) after growth under racemizing conditions (2 µL/mL).

An increase in average crystal size, consistent with growth, can be observed from the resulting scanning electron micrographs (Figure S-14). Moreover, initially present satellite crystals could no longer be observed after growth, possibly due to dissolution, growth, coalescence and/or ripening phenomena. Also note the change in the aspect ratio to more elongated crystals, which could indicate preferential growth or attachment on the tip, or be caused by the breaking of crystals due to collisions with other crystals, the vessel walls, or shearing.

I. Kinetic model based on the amplification factor

In our manuscript, we define the experimental amplification factor α:

$$\alpha = \frac{ee_\Delta}{ee_0} \tag{1}$$

where ee_Δ is the enantiomeric excess of the grown material during crystallization and ee_0 is the enantiomeric excess of the seed material. Here, we will use this definition to derive a simple model for deracemization kinetics under the assumption that nonlinear amplification ($\alpha > 0$) only occurs during crystal growth. The model can be shown to lead to an exponential relationship under realistic assumptions, a hallmark of all deracemization kinetics. The applicability of this model will then be demonstrated for the two major forms of CIET: Temperature Cycling (TCID) and attrition-induced Ostwald ripening (Viedma Ripening). We conclude that the mechanistic framework and experimental amplification factor are in agreement with current theory and kinetic experiments.

Derivation of a kinetic model based on the experimental amplification factor

We start by recalling the definition of the enantiomeric excess (ee):

$$ee = \frac{[R]-[S]}{[R]+[S]} = \frac{[R]-[S]}{m} \tag{2}$$

where [X] is the mass fraction of X and m is the total mass fraction of both R and S enantiomers combined. We can than note that

$$ee \cdot m = [R]-[S] \tag{3}$$

so that the enantiomeric excess after crystal growth (ee_P) is given by

$$ee_P = \frac{ee_0 \cdot m_0 + ee_\Delta \cdot m_\Delta}{m_0 + m_\Delta} \tag{4}$$

This equation (eq. 4) is described, in altered form, in the main text of the manuscript for the calculation of ee_Δ from experimental samples.

Deracemization consists of crystal growth and crystal dissolution steps. By using eq. 1 (so that $ee_\Delta = \alpha \cdot ee_0$), we can write for crystal growth:

$$ee_{P,\text{growth}} = \frac{\alpha \cdot ee_0 + \frac{m_0}{m_\Delta} \cdot ee_0}{1 + \frac{m_0}{m_\Delta}} = \left(\frac{\alpha + \frac{m_0}{m_\Delta}}{1 + \frac{m_0}{m_\Delta}}\right) \cdot ee_0 \tag{5}$$

To describe the effect of crystal dissolution, we can adapt equation 4. First, we set $\alpha = 0$ (since $ee_\Delta = 0$ assumes no amplification during dissolution). To account for the mass balance, we further substitute $m_0 \to m_0 + m_\Delta$ and $m_\Delta \to - m_\Delta$ so that

$$ee_{P,\text{dissolution}} = \left(\frac{\frac{m_0+m_\Delta}{m_\Delta}}{\frac{m_0+m_\Delta}{m_\Delta} - 1}\right) \cdot ee_0 \tag{6}$$

In the combined cycle, we move from crystal growth to crystal dissolution (and back again), so that we can combine eq's 5 and 6 by using

$$ee_{P,\text{cycle}} = \frac{ee_{P,\text{growth}} \cdot ee_{P,\text{dissolution}}}{ee_0} = \left(\frac{\alpha + \frac{m_0}{m_\Delta}}{1 + \frac{m_0}{m_\Delta}}\right) \cdot \left(\frac{\frac{m_0+m_\Delta}{m_\Delta}}{\frac{m_0+m_\Delta}{m_\Delta} - 1}\right) \cdot ee_0 = \left(\frac{\alpha \cdot m_\Delta}{m_0} + 1\right) \cdot ee_0 \tag{7}$$

Now we can define the deracemization process as a number of these cycles (n) for which the enantiomeric excess of the solid phase at the end of each cycle ee_n is given by

$$ee_n = \left(\frac{\alpha \cdot m_\Delta}{m_0} + 1\right) \cdot ee_{n-1} \tag{8}$$

, which (if we consider α invariant with n) is a geometric sequence so that

$$ee_n = ee_0 \cdot \left(\frac{\alpha \cdot m_\Delta}{m_0} + 1\right)^n \tag{9}$$

In reality, the question naturally rises whether α is indeed constant between cycles. Our experiments have shown, however, that his assumption is reasonable and α does not very much depend on ee_0, barring theoretical constraints (α is limited to $1/ee_0$).

For application in describing deracemization kinetics, we can define a typical cycle time τ so that $t = n \cdot \tau$. This leads to the time-dependent deracemization kinetics as

$$ee(t) = ee(0) \cdot \left(\frac{\alpha \cdot m_\Delta}{m_0} + 1\right)^{\frac{t}{\tau}} \tag{10}$$

To demonstrate the kinetic model based on the experimental amplification factor, we will show a fit to the two main types of experimental deracemization data: Temperature Cycling (TCID) and Viedma Ripening (attrition-enhanced).

<u>Application to Temperature Cycling</u>

Mazzotti and co-workers performed an optimization study on the temperature cycling of clopidogrel precursor BCA (compound **1** in our manuscript).[40] They measured the kinetics of temperature cycles while they varied the cooling rate during the crystal growth (cooling) step. After we calculated the m_0 and m_Δ based on the given experimental details and solubility data in [40], we were able to fit the kinetic data presented by the Mazzotti group to our kinetic model (eq. 9) based on the experimental amplification factor. The results (points) and our fit (lines) are shown in Figure S-15. There is a good agreement between our fit and the experimental results, implying that the kinetic model based on the experimental amplification factor is reasonable for deracemization via temperature cycles. This is in line with the proposed exponential behaviour of the process in literature. Moreover, the fitted values for the amplification factor, varying from 0.2 to 0.3 (depending on the cooling rate), are reasonable based on our own experimental results. The actual amplification factor will probably be higher, since the model assumes constant α, independent of ee and the changing crystallization kinetics, and fitting using the exponential in that way does not take into account the theoretical limit of $\alpha_n \leq 1/ee_{n-1}$.

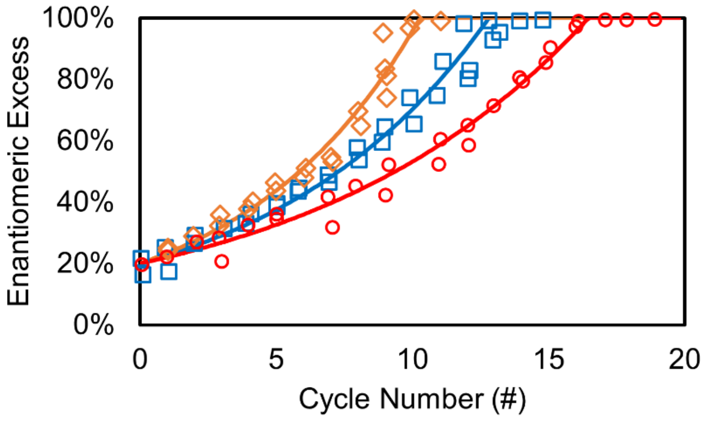

Figure S-15. Fitting of temperature cycling data from Breveglieri et al. based on eq. 9 using actual values of ee_0, m_Δ, and m_0. The resulting (fitted) amplification factors are in the range of 0.2 to 0.3. Original caption: Evolution of the BCA ee with the number of cycles at increasing cooling rate in going from experiment c3 (0.22 °C/min, orange markers) to c4 (0.43 °C/min, blue markers) to c5 (1.30 °C/min, red markers). Figure and caption adapted with permission from ref. 40. Copyright 2020 American Chemical Society.

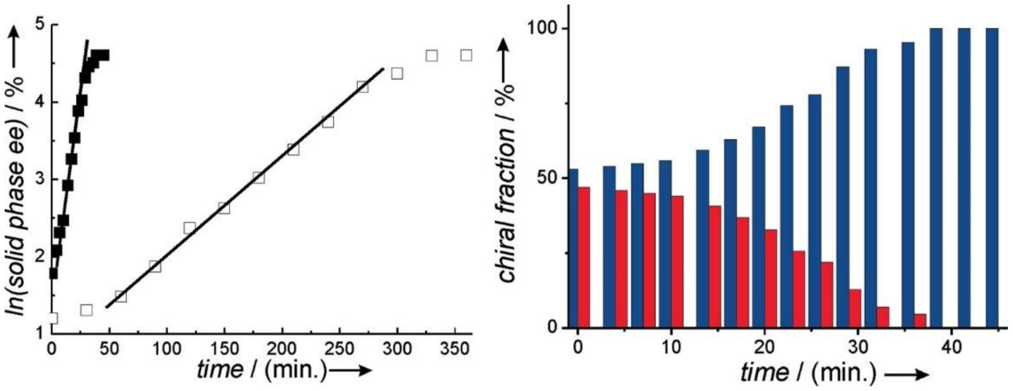

Figure S-16. Progression of the enantiomeric excess in the solid phase during the grinding-induced transformation on a 10-mL scale using a thermostatted ultrasonic cleaning bath (open symbols) and on a 400-mL scale using the bead mill (closed symbols). The straight lines are a fit of the data to eq. 11. The right graph shows the increase of the chiral purity in the solids of the latter experiment (blue (S)-**1**, red (R)-**1**). The initial ee values after dissolution were 3.40% for the ultrasonic and 5.96% in the beadmill experiment. Figure and caption reprinted with permission from ref. 59. Copyright 2010 American Chemical Society.

<u>Application to attrition-enhanced Ostwald ripening (Viedma Ripening)</u>

During Viedma Ripening, the concept of cyclic growth and dissolution fades, as there is a continuum of attrition that imposes continuous dissolution and growth. However, since crystal growth and crystal dissolution still appear as separate microscopic events, the model could still be applied to Viedma Ripening. Of course, under such conditions m_Δ gets a different meaning. Indeed, m_Δ will then pertain to the constant supersaturation imposed by the increased solubility of ground down crystals by virtue of the Gibbs-Thompson effect. This concept has been experimentally confirmed and it has been reported that the system is not in thermodynamic equilibrium, but in a kinetic equilibrium.[28] Also, the typical cycle time τ would better be re-interpreted via $1/\tau$ which then serves as a measure for the rates of crystal growth and dissolution under such grinding conditions (i.e. the frequency of microscopic growth and dissolution events). Both m_Δ and $1/\tau$ will, of course, vary with the strength of attrition and resulting kinetically stabilized crystal size distributions.

To apply the model to Viedma Ripening, we can rewrite eq. 10 by using the identity $x = \exp(\log(x))$ to yield

$$ee(t) = ee(0) \cdot \exp\left(\frac{\log\left(\frac{\alpha \cdot m_\Delta}{m_0} + 1\right)}{\tau} \cdot t\right) = ee(0) \cdot \exp(k \cdot t) \quad (11)$$

where k is a generic kinetic constant. Here, the deracemization rate constant k is formally defined as

$$k = \frac{\log\left(\frac{\alpha \cdot m_\Delta}{m_0} + 1\right)}{\tau} \approx \frac{\alpha \cdot m_\Delta}{m_0 \cdot \tau} \quad (12)$$

under the approximation that $\log(x+1) = x$ for small x (i.e. $x < ½$), which is generally valid in the slurry regime where m_0 is substantially bigger than m_Δ.

It has been well established that Viedma Ripening follows such exponential kinetics described by eq. 11. Amongst others, an example is that reproduced in Figure S-16 (also for compound **1**, the clopidogrel-precursor BCA).[59] Moreover this form has been theoretically advocated in other works as well and the description of k (eq. 12) predicts trends observed in other experimental work.[28,40,63] Therefore, a kinetic model based on the experimental amplification factor that we have introduced is in agreement with previous theoretical and experimental reports on deracemization via Viedma Ripening processes.
